# Antiplatelet effects of aspirin are not affected by the soluble guanylate cyclase activator cinaciguat (BAY 58-2667)

**DOI:** 10.1186/1471-2210-11-S1-P26

**Published:** 2011-08-01

**Authors:** Reiner Frey, Wolfgang Mueck, Michael Becka, Joern Kraetzschmar, Georg Wensing

**Affiliations:** 1 Clinical Pharmacology, Bayer HealthCare AG, Pharma Research Center, Wuppertal, Germany; 2 Global Biostatistics, Bayer HealthCare AG, Pharma Research Center, Wuppertal, Germany

## Introduction

Cinaciguat (BAY 58-2667) is an nitric oxide (NO)-independent and heme-independent soluble guanylate cyclase (sGC) activator. Cinaciguat preferentially activates sGC in its oxidized or heme-free state, when the enzyme is insensitive to its endogenous ligand NO and exogenous nitrovasodilators [1].

Endothelium-derived NO is one of the mechanisms by which platelet aggregation and thrombus formation is prevented by the intact blood vessel wall. Pharmacological stimulation of sGC in platelets correlates with inhibition of aggregation, platelet cGMP increase, prolongation of bleeding time and antithrombotic effects in vitro [2]. This protective mechanism may be impaired in vascular disease and impaired NO availability. Thus, sGC activators may have antithrombotic effects similar to endogenous NO. Indeed, cinaciguat potently inhibited platelet aggregation induced by the thromboxane mimic U46619 and collagen and prolonged rat-tail bleeding time up to 2-fold [3]. Thus, in vitro data suggested that coadministration of cinaciguat and aspirin may increase the antiplatelet effects of aspirin and could result in bleeding.

## Methods

This non-randomized, open-label trial was conducted from September to November 2001 in a single center in Germany. It was carried out in accordance with the Declaration of Helsinki and adhered to the International Conference of Harmonization good clinical practice guidelines and the German drug law (AMG). The study protocol was approved by the Ethics Committee of the North-Rhine Medical Council, Duesseldorf, Germany.

The study consisted of a 9-day treatment phase with 100 mg oral acetylsalicylic acid (aspirin) once daily administered in the morning in the fasting state and, after the last dose of aspirin, four sequential oral doses of 1.5 mg cinaciguat administered in the fasting state at 1-hour intervals. The first dose of cinaciguat was administered together with the last dose of aspirin. This cinaciguat dosing schedule was used to mimic a prolonged plasma concentration over time profile.

## Results

12 healthy men (mean age, 32.4 years, range, 23 - 42 years) were enrolled and completed the study according to protocol.

Low-dose aspirin treatment in healthy subjects resulted in the expected inhibition of platelet aggregation. 4 oral doses of 1.5 mg cinaciguat administered at 1-hour intervals did not significantly affect platelet aggregation induced by collagen and the thromboxane A2 mimetic U 46619 or bleeding time. These results are in line with the findings of a single dose escalation study.

Plasma pharmacokinetics of cinaciguat following 4 oral doses of 1.5 mg cinaciguat administered at 1-hour intervals showed pronounced inter-individual variability. There was no major pharmacokinetic interaction detected between aspirin and cinaciguat, compared with the data of a previous study, which applied the same dosing regimen.

## Conclusion

Coadministration of cinaciguat and aspirin did not reveal any pharmacodynamic interaction with respect of platelet aggregation or clinically relevant pharmacokinetic interaction.
